# Dental management in patients with antiplatelet therapy: A systematic review

**DOI:** 10.4317/jced.54079

**Published:** 2017-08-01

**Authors:** Luis-Miguel Sáez-Alcaide, Cristina Sola-Martín, Pedro Molinero-Mourelle, Victor Paredes-Rodríguez, Carmen Zarrias-Caballero, Gonzalo Hernández-Vallejo

**Affiliations:** 1 DDS. Postgraduate student. Department of Medicine and Oral Surgery, Faculty of Dentistry, Complutense University of Madrid; 2 DDS. Graduate student. Department of Medicine and Oral Surgery, Faculty of Dentistry, Complutense University of Madrid; 3 DDS, PhD. Assistant Professor. Department of Medicine and Oral Surgery, Faculty of Dentistry, Complutense University of Madrid; 4 DDS, MD, PhD. Associate Professor, Department of Medicine and Oral Surgery, Faculty of Dentistry, Complutense University of Madrid

## Abstract

**Introduction:**

Cardiovascular diseases are the most frequent cause of death in the Western world. Its treatment frequently needs therapy with antiplatelet agents, which increases the haemorrhage risk after oral surgical procedures. The aim of this study is to present a review on the dental management of the patients under antiplatelet treatment.

**Material and Methods:**

A systematic review was carried out following PRISMA recommendations including studies searched in Pubmed-Medline, Embase and Cochrane databases.

**Results:**

The current trend is to maintain the treatment during the surgical procedure, assuring a good control of the haemorrhage with local haemostatic measures. However, new antiplatelet drugs protocols are not firmly established.

**Conclusions:**

In spite of the existing recommendations, it is always advisable to consult with the internist or cardiologist of every patient before any intervention.

** Key words:**Antiplatelet, Oral Surgery, Exodontia, Dental Management.

## Introduction

Cardiovascular diseases were the main cause of mortality in USA in 2014 according to the National Institute of Statistics, being responsible of 167 deaths per 100.000 inhabitants. Among cardiovascular diseases, the ischemic ones (myocardial infarction, angina pectoris) were the first cause of death, followed by cerebrovascular accidents (CVA) (1). CVA represent the third cause of death in developed countries and they are one of the pathologies with major morbidity. About 85 % of the CVA are ischemic and, among these, 60 % have an atherothrombotic cause. Nowadays, the atheroesclerosis of the cerebral arteries is the most frequent cause of ischaemic stroke (2). Due to the high morbidity of these pathologies it is essential to know its causes, its prevention and its treatment. Among the pharmacological treatment of these diseases, antiplatelet agents have to be mentioned, used to prevent the development of atherothrombotic disease in individuals with risk factors (primary prophylaxis) as well as to avoid the relapse of cardiovascular and cerebrovascular events (secondary prophylaxis) in patients with established atherothrombotic disease (ischemic cardiopathy, peripheral arteriopathy and cerebrovascular disease), proving considerable reductions in the rates of mortality associated with the above mentioned pathologies (3).

An antiplatelet agent is described as a drug whose main effect is to inhibit the aggregation of thrombocytes and, therefore, the formation of a thrombus or clot inside the arteriovenous system. Any invasive or surgical procedure in the oral cavity involves intra and postoperative haemorrhage, being one of the most frequent emergencies for a dentist. This way, patients undergoing antithrombotic therapy show a higher haemorrhage risk. One treatment option is the interruption of the antithrombotic therapy eliminating this way the haemorrhage risk, nevertheless, the interruption implies a increased risk of cerebrovascular or cardiac thromboembolism. For this reason, it is necessary to manage patients undergoing these types of pharmacological treatments in order to minimize haemorrhagic as well as thromboembolism risks.

Thus, the aim of this study is to present antiplatelet drugs used for the treatment of these illnesses and also to establish guidelines for the approach of patients treated with antiplatelet drugs who are going to be treated with oral surgery procedures.

## Material and Methods

It was developed a protocol according to PRISMA recommendations for the realisation of systematic reviews (4) that included the following items:

- Population to study: The population of interest in this review included all those individuals in treatment with antiplatelet drugs undergoing oral surgery procedures.

- Type of intervention and comparison: The aim of the study was to compare and analyse the post-operatory bleeding after oral surgery in antiaggregated patients, interrupting or no their drug treatment prior to the surgical intervention.

- Results: The results according to the parameters used by each study to measure the post-operatory bleeding were analysed with the purpose of establishing a standardized protocol for these patients when implementing oral surgical procedures.

- Research Strategy: An electronic search was made in October of 2016 in the databases Pubmed-Medline, Embase and Cochrane, using the following search strategy: {Subject AND Adjective} {Subject: (antiplatelet OR novel antiplatelet OR clopidogrel OR prasugrel OR ticagrelor [Title]) AND Adjective: (oral surgery OR dental management OR dentistry [Title])}. From the beginning the search was not narrowed by language or by date. Due to the big number of articles found, only those written in English and Spanish from 2012 until now were selected.

- Inclusion criteria: 1) Series of patients under antiplatelet therapy; 2) Series of patients who underwent oral surgery; 3) These series had to include clinical data about the post-operatory bleeding after surgical procedure; 4) These series had to include pa-tients that continued with antithrombotic treatment and patients that interrupted it; 5) Longitudinal prospective and retrospective studies; 6) Transversal studies, 7) Clinical trials; 8) Reviews based on clinical studies with data about the management of this type of patients.

- Criteria of exclusion: 1) Series of patients without antiplatelet therapy; 2) Series that did not include clinical information on postoperative bleeding after the surgical procedure 3) Case reports; 4) Reviews not based on clinical studies; 5) Reviews that were not contributing relevant clinical information on the handling of these patients.

## Results

**Figure 1:**
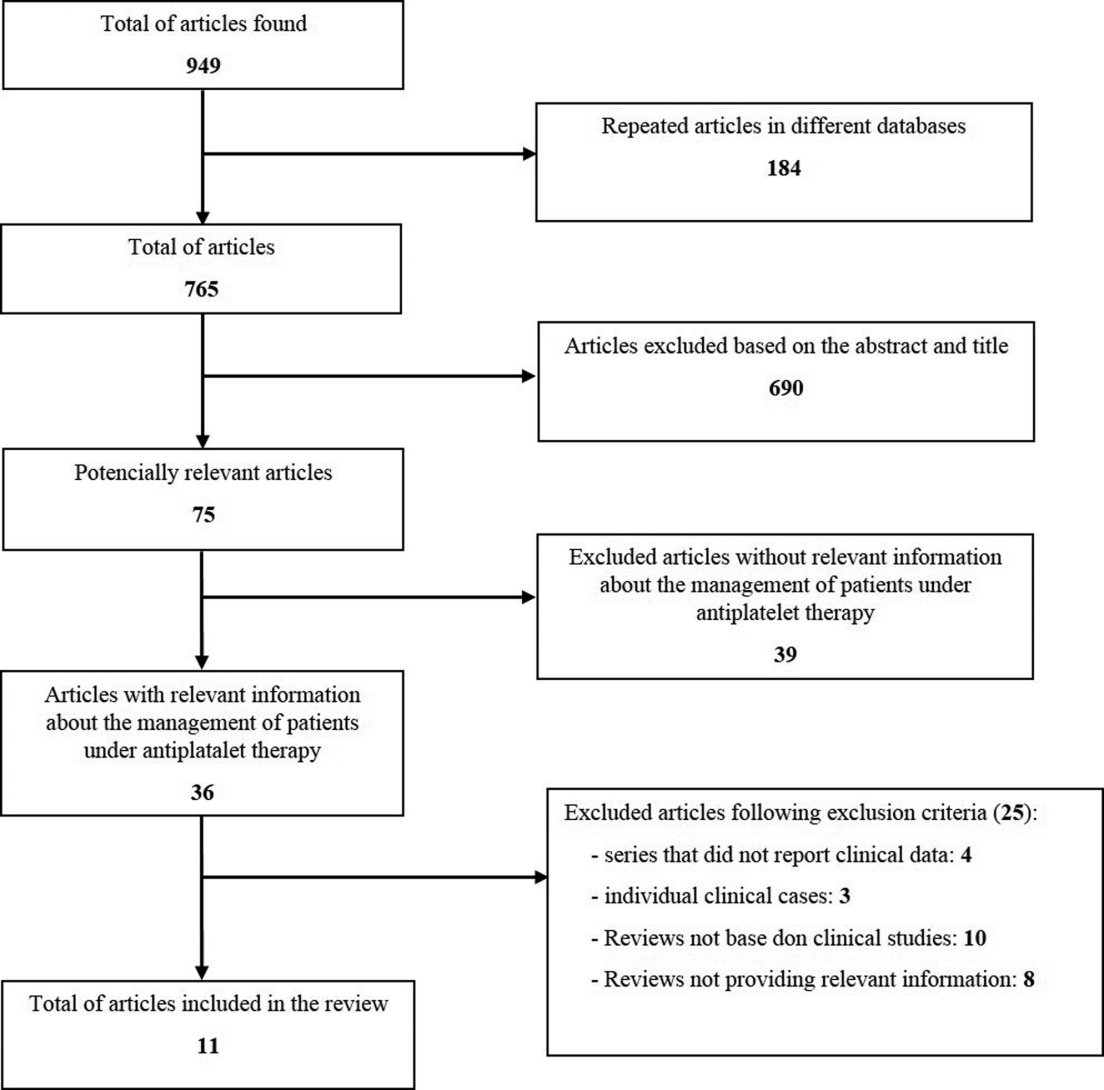
Flow Chart.

The initial search showed 949 studies whose title was related to the search patterns, turning out to be 765 after excluding the articles that were repeated in the different databases. Of this 765, 690 articles were excluded based on their title and summary, obtaining this way 75 potentially excellent articles. After the complete reading of these first potentially relevant 75 articles, only 36 articles, which include important information about the management in oral surgery of antiaggregated patients, were included. Next, 25 articles were excluded because of not fulfilling the inclusion criteria: 4 series were not providing clinical information on postoperative bleeding after the surgical procedure, 3 were case reports, 10 reviews were not based on clinical studies and 8 reviews did not provide clinical information on the handling of these patients. Thus, 23 articles were finally selected to perform the present review (Fig. 1).

-Quality of the chosen studies

Following the criteria of Harbour and Miller (5), it is possible to develop a classification of the levels of scientific evidence of the articles used in this study ( Table 1).

**Table 1:**
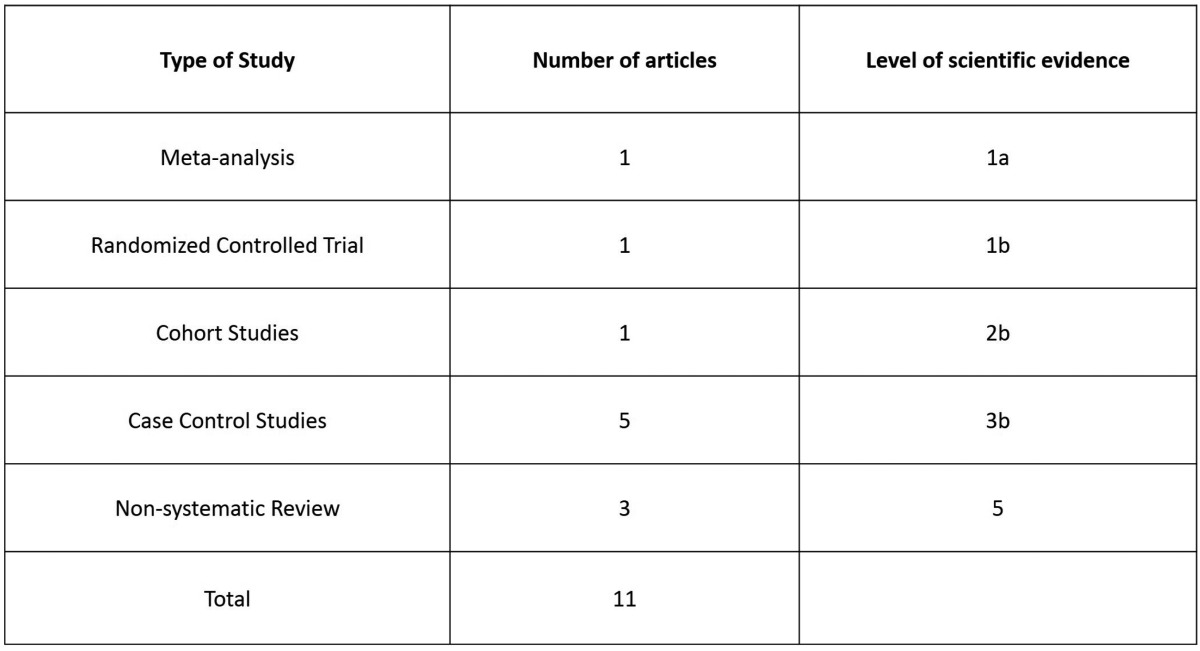
Type of articles included in the review.

These results reveal the first limitation of the present review, due to its large heterogeneity, both between the different studies as inside the studies themselves, without bearing in mind important variables for the analysis of the results. For example, the results are not compared by sex, nor by age, not even bearing in mind the rest of concomitant systemic pathologies, which often, especially in hepatic pathologies, have a direct effect on the postoperative bleeding indexes. Also, every clinical study uses its own specific inclusion and exclusion criteria, which increases even more the heterogeneity of the sample. Also, not in all the studies the same type of surgical procedures is performed, nor by the same professionals, a fact which affects to a great extent the postoperative course of every patient.

Finally, no regulated and normalized method exists for the data acquisition on postoperative haemorrhagic complications after procedures of oral surgery. This supposes that the complications quoted in every study have been reported without uniform criteria and are based on the subjectivity of every member of the group of investigators.

## Discussion

In order to decide to interrupt or maintain the antiplatelet treatment, it is necessary to assess in each patient the thrombotic risk, the bleeding risk and the invasiveness of each procedure.

-Assessment of the thromboembolic risk

One of the main challenges is to predict or try to prevent thromboembolic events. At present, some scales allow to quantify and classify the patients according to their risk of suffering a thromboembolism. One of the tools more used at present is the CHA2DS2-VASc (6) scale, which includes different parameters to evaluate the risk. ( Table 2)

**Table 2:**
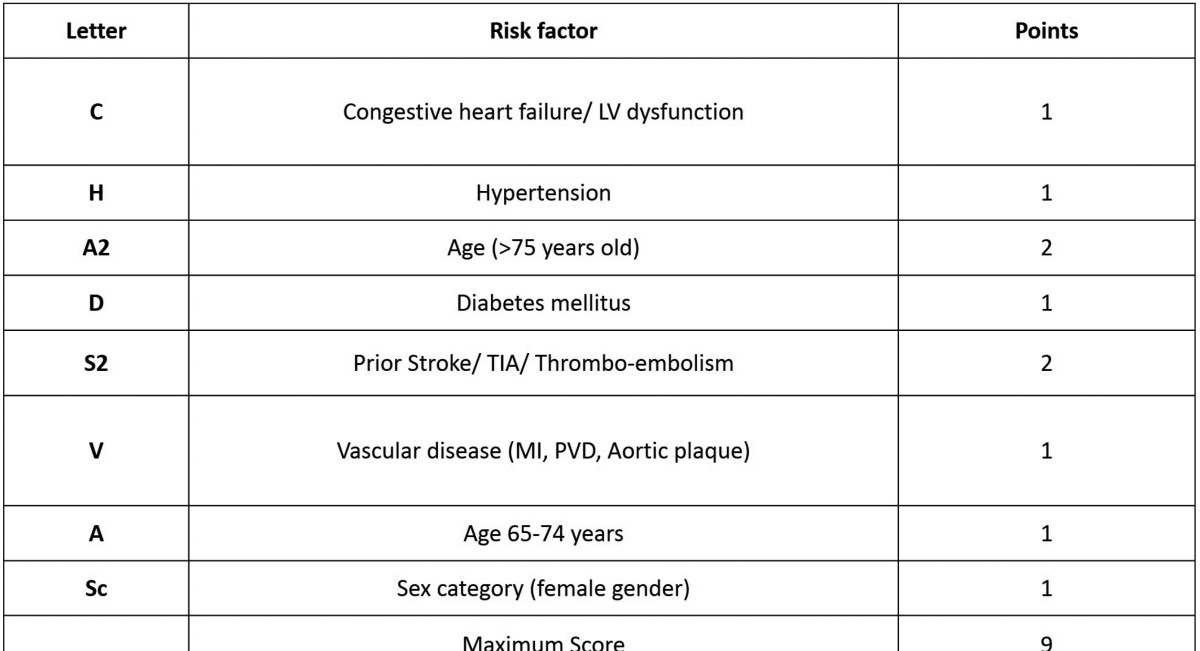
CHA2DS2-VASc Score for Stroke Risk in AFib (6).

This way, the maximum possible score to be achieved is 9. Based on these measurements, a score under 1 implies low risk, which means there is no need of antithrombotic treatment or treatment with antiplatelet drugs; a score of 1 involves moderate risk and requires treatment with antiplatelet or anticoagulant drugs and a score over 2 requires treatment with anticoagulant drugs (7).

-Assessment of the haemorrhagic risk

The most test to measure the antithrombotic effect of the antiplatelet therapy are aggregometry, thromboelastography, flow cytometry, monitoring of TXA2 synthesis, the VerifyNow® or the Platelet Function Analyzer. Usually it is not necessary to continuously monitorize the patients who take antiplatelet drugs (8). As a complementary method, the HAS-BLED scale, proposed in 2010 by the European Society of Cardiology (7), is very useful to assess the bleeding risk of each patient. In this scale, a score of 0 is considered low risk, 1-2 medium risk and ≥3 high risk (7,9) ( Table 3).

**Table 3:**
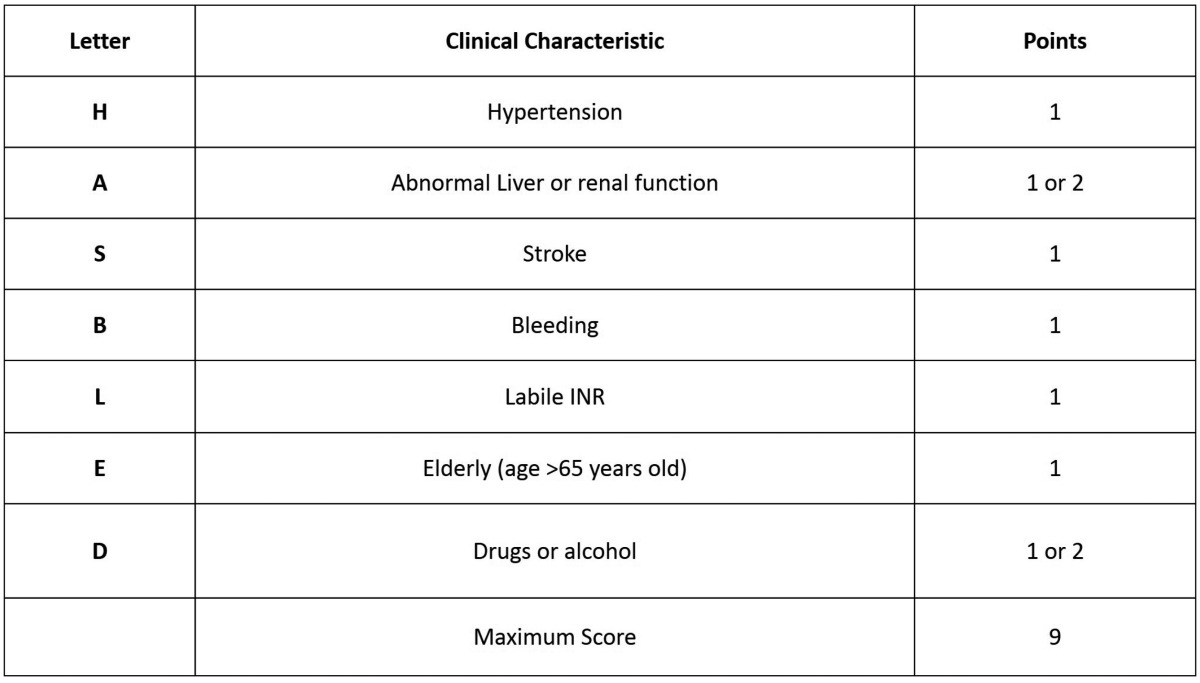
HAS- BLED Score for Major Bleeding Risk (7).

-Dental procedure invasiveness

At present no consensus exists about which dental procedures are considered to have low or high bleeding risk, since every author proposes a particular classification. Uniting the information of several studies (10-12) it is possible to establish the following classification.

Regardless of the antithrombotic treatment the patient receives, it is recommended to apply all the local haemostatic measures known to reduce as much as possible the bleeding risk and ensure a correct haemostasis ( Table 4).

**Table 4:**
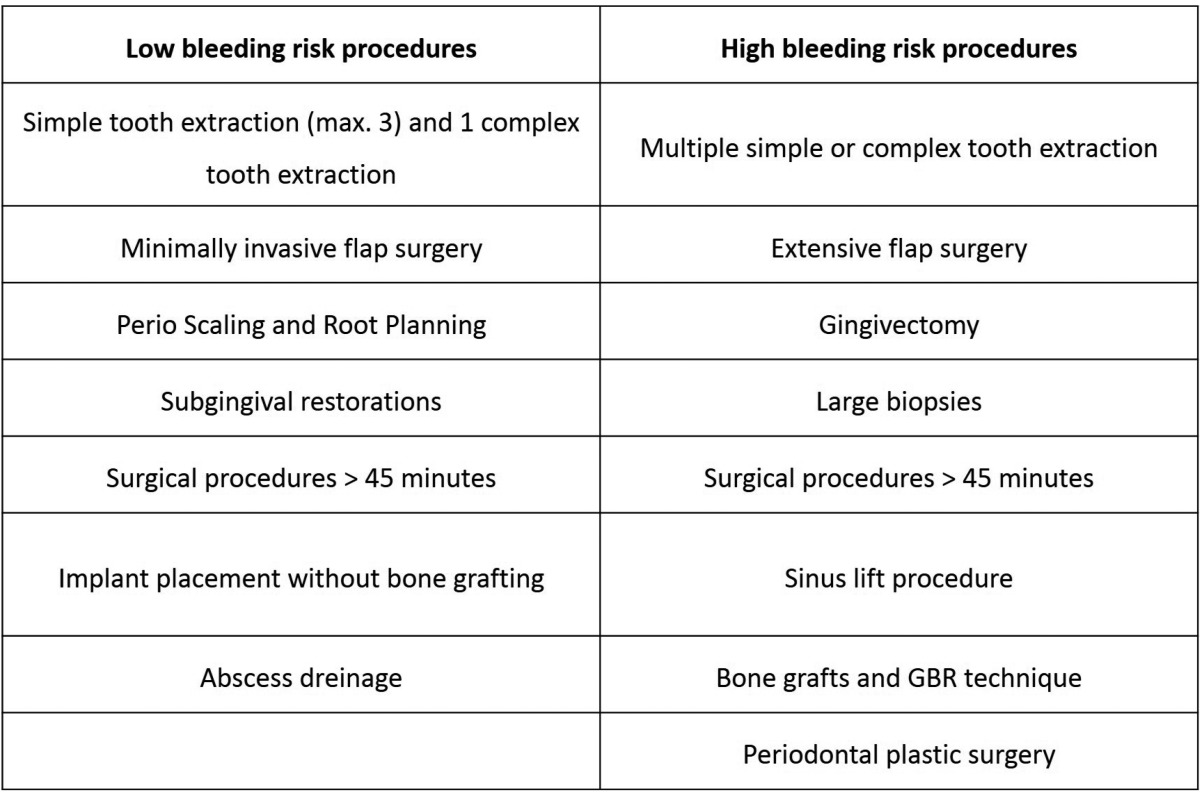
Authors’ proposal of classification according to the risk of the surgical procedure.

-Acetylsalicylic Acid

Nowadays, most publications state it is not necessary to stop medication with ASA in most oral surgical procedures. Authors as Lu, Johnston or Natwhani conclude in their studies there is no need to retire ASA medication to implement oral surgery procedures (13-15). In the study of Lu on 1,271 patients, the incidence of bleeding in the group with ASA medication was 1.1% vs. 0.7% in the control group (healthy patients without antiplatelet inhibitors nor anticoagulant treatment). These studies also evaluated postoperative haemorrhage in patients treated with two antiplatelets. They observed that double ASA+clopidogrel therapy produces more bleeding than monotherapy with ASA (4.4% of bleeding complications versus 0.7% in control group) but there is no need to interrupt this regimen for oral surgery procedures (13).

Postoperative haemorrhage following dental extractions in patients with ASA treatment compared with healthy patients without antiplatelet nor anticoagulant treatment was studied in a meta-analysis by Zhao and cols in 2015. From a total of 1,752 patients, 529 were in treatment with ASA and the rest were not. A longer postoperative bleeding time was observed in patients with ASA treatment but without statistically significant differences compared with the control group. Therefore, it was concluded that ASA treatment should not be interrupted at the time of dental extractions, provided that the right local haemostatic protocols are im-plemented (16).

On the other side, authors as Rubio-Alonso suggest that in cases of large maxillofacial surgeries or patients with very low risk of thromboembolism, ASA treatment can be suspended 7 days before the surgery (10,17).

-Clopidogrel

There are not many current studies available assessing postoperative bleeding after oral surgery in patients treated just with clopidogrel, but a higher number of publications exist assessing treatment with ASA+clopidogrel. However, there are studies as the one by Eichron and cols. which assess bleeding complications after oral and maxillofacial surgery in patients treated with clopidogrel versus a control group. In a group of 650 patients, 127 were being treated with clopidogrel. They presented statistically significant differences in postoperative bleeding complications, all of which were easily handled with local haemostatic protocols (18).

The retrospective study carried out by Lu and cols shows no significant differences in bleeding rate between ASA, clopidogrel and ASA+clopidogrel (13).

Dezsi and cols studied postoperative bleeding in patients treated with ASA+clopidogrel, concluding that with proper haemostatic protocols it is not necessary to interrupt the use of both drugs to keep bleeding under control (19). Similar conclusions were re-ported by authors as Dudek, Bajkin and Olmos–Carrasco (20-22).

Even in more traumatic surgeries that invade the bone, it has been seen that there is no need to stop using clopidogrel. The retrospective study by Grobe and cols. in 341 patients, in the experimental group (patients treated with ASA+clopidogrel) showed a 3.3% of bleeding complications compared to the control group, which showed 0.7% (23).

Therefore, all these authors do not recommend stopping treatment with this drug before surgery if correct haemostatic protocols are implemented

On the other side, authors such as Rubio-Alonso suggest that in cases of large maxillofacial surgeries or in cases with very low risk of thromboembolism, the intake of clopidogrel can be interrupted between five and seven days before the surgery and started again 24 hours after the treatment (10,17).

-Prasugrel and Ticagrelor

The existing current evidence in the literature referring to these two drugs is much more limited due to the lack of strong studies supporting consensual and well established protocols. Their producers recommend suspending treatment with these drugs 7 days before surgical procedures. Even so, due to the fact that oral surgery tends to be considered as a minor surgery, this recommenda-tion is not very useful from a clinical point of view (24).

Johnson states that the limited existing scientific evidence on these drugs does not allow discontinuing systematically the treatment with prasugrel or ticagrelor when performing oral surgical procedures (25). The study by Deszi shows that when performing dental extractions bleeding lasted more in patients treated with prasugrel, but did not find statistically significant differences in postoperative bleeding rates, concluding that there is no need to suspend treatment with prasugrel when performing oral surgery (19).

In 2014, Olmos-Carrasco and cols. carried out a randomized controlled prospective multicenter study in which they studied pos-toperative bleeding complications (after dental extractions in 181 patients in treatment with two blood thinners, some of them in therapy with ASA+prasugrel). They observed an 8.3% rate of postoperative bleeding complications, all of them solved in less than 30 minutes with conventional haemostatic protocols. According to this, it was concluded that it was not necessary to stop antiplatelet therapy (20).

Most publications and protocols with enough scientific evidence to be implemented clinically are those referring to ASA and/or clopidogrel. Thus, most studies conclude that it is not necessary to stop antiplatelet therapy in oral surgery procedures provided that proper haemostatic protocols are followed (13,16,18,19).

## Conclusions

1. Regardless of the procedure to be performed, the medical general condition is more important than the dental needs.

2. Whatever procedure is to be implemented, it is advisable to take into account all the local haemostatic measures known.

3. With respect to conventional antiplatelet drugs, the most common tendency is not to withdraw the drug provided as long as postoperative bleeding is controlled.

4. In the case of the new antiplatelet drugs there is not enough scientific evidence available as to establish strict protocols. Therefore, more clinical studies with more patients and longer follow-up are needed to derive more accurate and reliable conclusions.
